# Age‐specific prevalence of human papillomavirus and abnormal cytology at baseline in a diverse statewide prospective cohort of individuals undergoing cervical cancer screening in Mississippi

**DOI:** 10.1002/cam4.4340

**Published:** 2021-11-03

**Authors:** Megan A. Clarke, Carolann Risley, Mary W. Stewart, Kim R. Geisinger, Laree M. Hiser, Jody C. Morgan, Kenyata J. Owens, Krishna Ayyalasomayajula, Rhonda M. Rives, Ashish Jannela, Dianne E. Grunes, Lei Zhang, Mark Schiffman, Sarah Wagner, Joseph Boland, Sara Bass, Nicolas Wentzensen

**Affiliations:** ^1^ Clinical Genetics Branch Division of Cancer Epidemiology & Genetics National Cancer Institute Rockville Maryland USA; ^2^ School of Nursing University of Mississippi Medical Center Jackson Mississippi USA; ^3^ Walter Reed National Military Medical Center Armed Forces Joint Pathology Center Bethesda Maryland USA; ^4^ Center for Informatics & Analytics University of Mississippi Medical Center Jackson Mississippi USA; ^5^ Department of Pathology University of Mississippi Medical Center Jackson Mississippi USA; ^6^ Office of Health Data & Research Mississippi State Department of Health Jackson Mississippi USA; ^7^ Cancer Genomics Research Laboratory Division of Cancer Epidemiology and Genetics NCI Bethesda Maryland USA

**Keywords:** cervical cancer, human papillomavirus, Mississippi, natural history, racial differences

## Abstract

**Background:**

Mississippi (MS) has among the highest rates of cervical cancer incidence and mortality in the United States, with disproportionately higher rates among Blacks compared to Whites. Here, we evaluate the prevalence of high‐risk human papillomavirus (HPV) and abnormal cytology in a representative baseline sample from a diverse statewide cohort of individuals attending cervical screening in MS from the STRIDES Study (STudying Risk to Improve DisparitiES in cervical cancer).

**Methods:**

We included individuals aged 21–65 years undergoing screening at the University of Mississippi Medical Center (UMMC) and the Mississippi State Department of Health (MSDH) from May to November 2018. We calculated age‐specific HPV prevalence, overall and by partial HPV16/18 genotyping, and abnormal cytology by race.

**Results:**

A total of 6871 individuals (mean age 35.7 years) were included. HPV prevalence was 25.6% and higher in Blacks (28.0%) compared to Whites (22.4%). HPV prevalence was significantly higher in Blacks aged 21–24 years (50.2%) and 30–34 years (30.2%) compared to Whites in the same age groups (32.1% and 20.7%; p < 0.0001, respectively). The prevalence of high‐grade cytologic abnormalities, a cytologic sign of cervical precancer, peaked earlier in Blacks (ages 25–29) compared to Whites (35–39). For comparison, we also analyzed HPV prevalence data from the National Health and Nutrition Examination Survey (NHANES, 2013–2016) and observed similar racial differences in HPV prevalence among women aged 21–24 years.

**Conclusions:**

Our findings suggest that Blacks undergoing cervical cancer screening in MS have higher prevalence of other high‐risk 12 HPV types at younger ages and experience an earlier peak of high‐grade cytologic abnormalities compared to Whites.

## Graphical abstract

We evaluated the age‐specific prevalence of human papillomavirus (HPV) and abnormal cytology in a baseline sample from a large, diverse statewide screening cohort in Mississippi. Overall, HPV prevalence was high (25.6%), and varied by race, with Blacks having significantly higher prevalence of other high‐risk 12 HPV types at younger ages compared to Whites, corresponding to an earlier peak of high‐grade cytologic abnormalities.

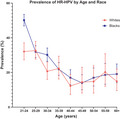

## 1 INTRODUCTION

Cervical cancer screening has led to dramatic reductions in cervical cancer incidence and mortality over the past decades in the United States; however, thousands continue to develop and die from cervical cancer each year, with significant disparities observed by geographic region as well as race and ethnicity.^1^, ^2^ In Mississippi (MS), which has one of the highest burdens of cervical cancer in the United States, Blacks have higher cervical cancer mortality compared to Whites (5.0 vs. 2.8 per 100,000, 2013–2017).^3^ The underlying causes of these disparities are likely multi‐faceted, involving factors related to access to care and potentially also factors related to human papillomavirus (HPV) natural history.

While the causal role of HPV in cervical carcinogenesis is well‐established and thought to be universal across settings,^4^ age‐specific prevalence of HPV infection, precancer, and cancer varies across populations, related to behavioral factors such as average age of sexual initiation and biologic factors such as immunologic control of infections.^5^ In many settings, including the United States, HPV prevalence peaks shortly after sexual debut and declines with increasing age, corresponding to a decrease in exposure to new sexual partners and some level of acquired immunity to repeat HPV infection.^5^ This model guides recommendations that routine vaccination should be administered to adolescents aged 11 or 12 years,^6^, ^7^ and that HPV screening should not occur before age 25 years due to the high prevalence of benign HPV infections and the low prevalence of cervical precancers in younger individuals.^8^, ^9^ HPV genotype prevalence in precancers and cancers varies by race and ethnicity. For example, HPV35, a carcinogenic type not included in current vaccines, is more prevalent among individuals of African descent compared to Whites.^10^, ^11^, ^12^, ^13^, ^14^

As HPV‐based cancer prevention approaches are increasingly being implemented, population‐based studies of HPV prevalence in regions like MS are necessary to better understand how these tools will perform in racially diverse populations with high rates of cervical cancer. To address this gap, we designed the STRIDES study (STudying Risk to Improve DisparitiES in cervical cancer in MS) which includes over 30,000 individuals undergoing cervical cancer screening throughout the state of MS.^15^ Here, we evaluate the age‐specific prevalence of high‐risk HPV infection in a representative subset of this population including individuals aged 21–65 years, with a particular focus on racial differences. We put our findings in context with nationally representative data from the National Health and Nutrition Examination Survey (NHANES, 2013–2016).

## 2 MATERIALS AND METHODS

### 2.1 Study population

Data included in this study are from a representative baseline sample of a large statewide cohort study, STRIDES. Briefly, STRIDES includes individuals undergoing cervical cancer screening and management at the University of Mississippi Medical Center (UMMC) and the Mississippi State Department of Health (MSDH). UMMC is the sole academic medical center in the state, and all cervical pathology samples from MSDH clinics are sent to UMMC. Screening and management data are obtained from electronic health records (EHRs). Discard specimen collection began in May 2018, and follow‐up is ongoing. The current cross‐sectional study is a representative subset of the STRIDES cohort, including all individuals aged 21–65 years who underwent screening from 8 May 2018 to 26 November 2018. The Institutional Review Boards at UMMC and MSDH approved this protocol; a HIPAA waiver of consent was granted.

### 2.2 Liquid‐based cytology

All specimens are processed and interpreted in the UMMC Department of Pathology. Liquid‐based cytology (Pap) testing is conducted using the ThinPrep Pap 2000 System (Hologic^®^). Specimens are processed and prescreened with automated image analysis, followed by full screening by the cytotechnologist prior to final cytologic interpretation by a cytopathology fellowship‐trained pathologist. Cytologic interpretation occurs without prior knowledge of the HPV result. Cytology results are interpreted using the Bethesda 2014 terminology^16^ and classified as negative for intraepithelial lesion or malignancy (NILM), atypical squamous cells of undetermined significance (ASC‐US), low‐grade squamous intraepithelial lesion (LSIL), atypical squamous cells cannot exclude high grade (ASC‐H), or high‐grade squamous intraepithelial lesion (HSIL). Glandular lesions and atypical endometrial cells are rare in this population and therefore grouped together as “other.”

### 2.3 HPV testing

Two HPV assays were included in this study: cobas 4800 for specimens with clinical HPV testing and the TypeSeq assay for specimens not sent for clinical HPV testing, described in detail below. At MSDH, screening transitioned from primary cytology with ASC‐US triage with reflex HPV testing to HPV and cytology co‐testing on 1 July 2018, for those aged 30–65 years. Individuals under 30 years of age undergo primary cytology screening with ASC‐US triage. UMMC clinics predominantly perform co‐testing among individuals aged 30 years and older and primary cytology with HPV ASC‐US triage for those under age 30; however, some UMMC providers continue to perform screening using primary cytology regardless of age. MSDH and UMMC specimens that were sent for clinical HPV testing were tested using the Roche Diagnostics cobas 4800^®^ HPV genotyping test (Roche Molecular Systems) (Roche, 2018). The assay provides type‐specific identification of types 16 and 18 and pools other high‐risk HPV genotypes: 31, 33, 35, 39, 45, 51, 52, 56, 58, 59, and 68. HPV66 is also assayed although its inclusion is of questionable value.^17^ Together, these 12 types are grouped as “other HR12.”

Specimens that were not sent for clinical HPV testing were sent to the National Cancer Institute's Cancer Genomics Research Laboratory for testing with TypeSeq.^18^, ^19^ TypeSeq detects 51 HPV types (HPV3, 6, 11, 13, 16, 18, 26, 28, 30, 31, 32, 33, 34, 35, 39, 40, 42, 43, 44, 45, 51, 52, 53, 54, 56, 58, 59, 61, 62, 66, 67, 68, 69, 70, 71, 72, 73, 74, 76, 81, 82, 83, 84, 85, 86, 87, 89, 90, 91, 97, and 114). Specimens not meeting minimum human and/or HPV read thresholds were reported as “failed to amplify.” To generate results compatible with cobas, we classified the same 14 types as high‐risk and also separately analyzed types 16 and 18 versus other HR12. A total of 3123 (45.5%) individuals were tested with cobas 4800, 3634 (52.9%) were tested with TypeSeq, and 369 (5.4%) samples were not collected or sent for HPV testing. A total of 34 samples tested by TypeSeq failed to amplify (0.9%).

### 2.4 Demographics and co‐variates

We collected demographic information including age and race from the EHR based on the intake information recorded from the patient at the screening visit. Race and ethnicity included the following categories from the EHR: White or Caucasian (“White”), Black or African American (“Black”), American Indian or Alaska Native, Asian, Native Hawaiian or Other Pacific Islander, Multiracial, Other Race, and includes Patient Refused or Unknown. Due to low sample size, American Indian or Alaska Native, Choctaw Indian, Asian, Native Hawaiian, or Other Pacific Islander, were collapsed into one category (“Other Race”). Age was categorized in 5‐year groups from 21 to 24 years to 60+ years. For some analyses where numbers were sparse, we combined older age groups as 40–49, 50–59, and 60+ years.

### 2.5 Statistical analyses

We included data from the baseline visit, defined as the first observed screening record in the EHR as of May 2018. If HPV testing data were missing from the baseline visit but collected in a subsequent visit within 6 months, we used data from that second visit (*n* = 36). We used descriptive statistics to summarize baseline characteristics and chi‐square and one‐way ANOVA testing to evaluate differences in baseline characteristics by race. Among Whites and Blacks where we had large enough sample size, we calculated the proportion and 95% confidence intervals (CIs) of individuals who tested positive for high‐risk HPV, overall and by HPV16/18 versus other HR12, by race, and stratified by age group. Among all individuals irrespective of HPV results, we evaluated the distribution (proportions and 95% CIs) of cytology by race, stratified by age group, grouping together ASC‐US and LSIL as low‐grade cytologic abnormalities and ASC‐H and HSIL as suggestions of underlying cervical precancer. We also computed proportions of overall HPV positivity by continuous age and race using a 5‐year moving average for individuals aged 21–65 years of age.

To contextualize our findings within the US population we analyzed HPV prevalence data from NHANES using the most recent consecutive cycles (2013–2016). Briefly, NHANES is an ongoing series of population‐based cross‐sectional surveys conducted by the National Center for Health Statistics at the Centers for Disease Control (CDC). The survey consists of a household interview and a physical examination in a mobile examination center. Cervicovaginal samples were self‐collected at a mobile examination center and sent to the CDC laboratory for HPV testing and genotyping with linear array.^20^ We grouped HPV genotypes as HPV16/18 and other HR12 and calculated prevalence estimates by applying sample weights to account for selection probabilities and non‐response. We used Taylor series linearization to calculate standard errors.

All reported *p* values were two‐sided, and a *p* value <0.05 was considered significant. Statistical analyses were conducted using STATA/SE, version 16.0.

## 3 RESULTS

### 3.1 Population characteristics

A total of 6871 individuals screened between May 2018 and November 2018 at either MSDH (*n* = 4624; 67.3%) or UMMC (*n* = 2247; 32.7%) were included in this analysis. The mean age of all individuals was 35.7 years and varied by race, with Whites being slightly older on average compared to the other two groups (Table 1). Among all individuals, the prevalence of HPV infection was 25.6%, with Blacks having the highest prevalence (28.0%), followed by Whites (22.4%), and those classified as Other race (21.0%). A total of 403 individuals (5.9%) were missing HPV results; these individuals were slightly older (mean age 40.8 years), less likely to be “Other” race (6.5%), and more likely to have inadequate cytology results (4.5%; data not shown) compared to the overall study population. The prevalences of HPV16 and HPV18 were 3.0% and 1.7%, respectively, in the total population and did not vary by race. The prevalence of other HR12 HPV types was 24.9% among all individuals and was higher among Blacks (27.5%) compared to other racial groups (21.3% in Whites and 20.0% in Other). Overall, 82.5% of the study population had normal cytology (NILM) results, with individuals classified as Other race having higher prevalence of normal cytology (86.6%) compared to Whites (83.7%) and Blacks (81.1%).

**TABLE 1:** Baseline characteristics of individuals with cytology and HPV testing overall and by race

	Total	Whites	Blacks	Other
Total	6871 (100.0)	1803 (26.2)	4192 (61.0)	876 (12.8)
Screening site, *n* (%)				
MSDH	4624 (67.3)	1109 (61.5)	2759 (65.8)	756 (86.3)
UMMC	2247 (32.7)	694 (38.5)	1433 (34.2)	120 (13.7)
Mean age (years, (SD))	35.7 (12.9)	36.7 (14.0)	35.7 (12.9)	33.5 (10.1)
Age group, *n* (%)				
21–24	1512 (22.0)	388 (21.5)	940 (22.4)	184 (21.0)
25–29	1337 (19.5)	355 (19.7)	812 (19.4)	170 (19.4)
30–34	1032 (15.0)	274 (15.2)	588 (14.0)	170 (19.4)
35–39	856 (12.5)	173 (9.6)	536 (12.8)	147 (16.8)
40–44	566 (8.2)	145 (8.0)	328 (7.8)	93 (10.6)
45–49	450 (6.6)	105 (5.8)	294 (7.0)	51 (5.8)
50–54	357 (5.2)	100 (5.6)	234 (5.6)	23 (2.6)
55–59	309 (4.5)	98 (5.4)	193 (5.6)	23 (2.6)
60+	452 (6.6)	165 (9.1)	267 (6.4)	20 (2.3)
High‐risk HPV, *n* (%)				
Positive	1761 (25.6)	403 (22.4)	1174 (28.0)	184 (21.0)
Negative	4707 (25.6)	1286 (71.3)	2755 (65.7)	666 (76.0)
Missing	403 (5.9)	114 (6.3)	263 (6.3)	26 (3.0)
Any HPV16, *n* (%)	195 (3.0)	58 (3.4)	119 (3.0)	19 (2.2)
Any HPV18, *n* (%)	109 (1.7)	27 (1.6)	72 (1.8)	10 (1.2)
Any other HR12, *n* (%)	1609 (24.9)	359 (21.3)	1080 (27.5)	170 (20.0)
Cytology, *n* (%)				
Inadequate	83 (1.2)	26 (1.4)	48 (1.1)	9 (1.1)
NILM	5667 (82.5)	1509 (83.7)	3399 (81.1)	759 (86.6)
ASC‐US	506 (7.4)	117 (6.5)	336 (8.0)	53 (6.1)
LSIL	453 (6.6)	105 (5.8)	302 (7.2)	46 (5.3)
ASC‐H	42 (0.6)	11 (0.6)	27 (0.6)	4 (0.5)
HSIL	99 (1.4)	27 (1.5)	67 (1.6)	5 (0.6)
Other	21 (0.3)	8 (0.4)	13 (0.3)	0 (0.0)

Abbreviations: ASC‐H, atypical squamous cells cannot exclude high grade; ASC‐US, atypical squamous cells of undetermined significance; HSIL, high‐grade intraepithelial lesion; LSIL, low‐grade intraepithelial lesion; MSDH, Mississippi State Department of Health; NILM, negative for intraepithelial lesion or malignancy; UMMC, University of Mississippi Medical Center.

As shown in Table 2, the distribution of cytology results by HPV positivity was similar between Whites and Blacks; however, among individuals with NILM cytology, Blacks were more likely to be HPV positive compared to Whites (23.0% vs. 18.3%, respectively, *p* < 0.0001). Differences in HPV positivity by race were not significant for other cytologic categories.

**TABLE 2:** Cytology and HPV results by race

	Whites (*N* = 1689)		Blacks (*N* = 3929)	
	HPV positive *N* (row%/col%)	HPV negative *N* (row%/col%)	HPV positive *N* (row%/col%)	HPV negative *N* (row%/col%)
Cytology				
Inadequate	1 (4.8/0.25)	20 (95.2/1.6)	3 (8.3/0.3)	33 (91.7/1.2)
NILM^a^	262 (18.3/65.0)	1167 (81.7/90.8)	733 (23.0/62.4)	2460 (77.0/89.3)
ASC‐US	42 (37.5/10.4)	70 (62.5/5.4)	156 (47.4/13.3)	173 (52.6/6.3)
LSIL	74 (79.6/18.4)	19 (20.4/1.5)	211 (76.5/18.0)	65 (23.6/2.4)
ASC‐H	5 (71.4/1.2)	2 (28.6/0.2)	16 (61.5/1.4)	10 (38.5/0.4)
HSIL	18 (85.7/4.5)	3 (14.3/0.2)	53 (88.3/4.5)	7 (11.7/0.3)
Other	1 (16.7/0.3)	5 (83.3/0.4)	2 (22.2/0.2)	7 (77.8/0.3)
Total	403 (23.9/100.0)	1286 (76.1/100.0)	1174 (29.9/100.0)	2755 (70.1/100.00)

Abbreviations: ASCH, atypical squamous cells cannot exclude high grade; ASCUS, atypical squamous cells of undetermined significance; HSIL, high‐grade intraepithelial lesion; LSIL, low‐grade intraepithelial lesion; NILM, negative for intraepithelial lesion or malignancy.

^a^ Among individuals with NILM cytology, Blacks were significantly more likely to be HPV positive compared to Whites, *p* < 0.0001.

### 3.2 Prevalence of HPV by age and race

**FIGURE 1:**
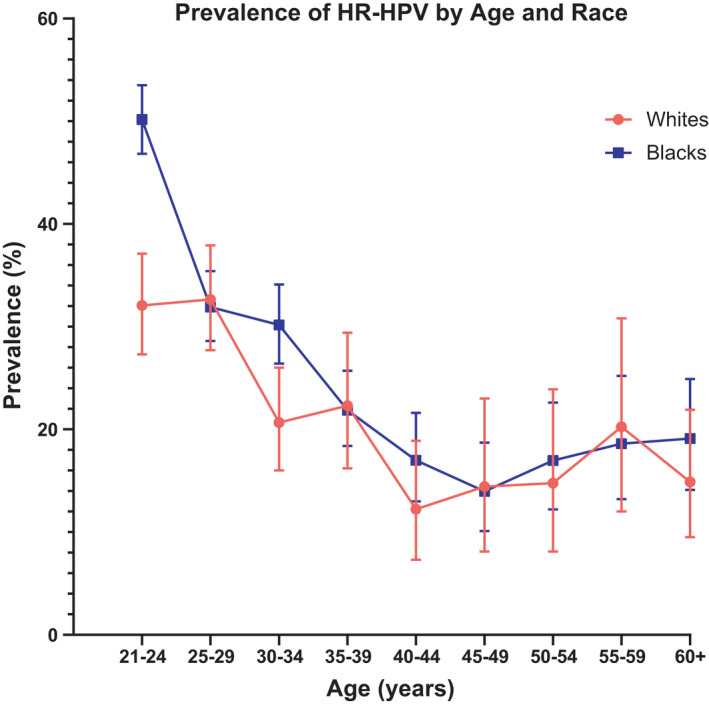
Prevalence of HR‐HPV by age and race. The prevalence (%) and 95% confidence intervals of high‐risk HPV infection including HPV types 16, 18, 31, 33, 35, 39, 45, 51, 52, 56, 58, 59, 66, and 68 (*y*‐axis) are plotted by age group (*x*‐axis). Prevalence curves are shown for Whites in red with filled circles and for Blacks in blue with filled squares. HPV, human papillomavirus; HR, high‐risk

As shown in Figure 1, the prevalence of HPV infection was significantly higher in Blacks aged 21–24 years (50.2%; 95% CI, 46.8–53.5%) and 30–34 years (30.2%; 95% CI, 26.4–34.1%) compared to Whites in the same age groups (32.1%; 95% CI 27.3–37.1% and 20.7%; 95% CI, 16.2–26.0%, *p* < 0.0001, respectively). The highest prevalence of HPV infection was observed around age 26 years (34.4%) among Whites, whereas the highest observed prevalence of HPV infection among Blacks occurred at age 21 years (51.3%) (Figure S1). In both groups, the prevalence of HPV infection declined with age.

### 3.3 Prevalence of other HR12 HPV and HPV16/18 by age and race

**FIGURE 2:**
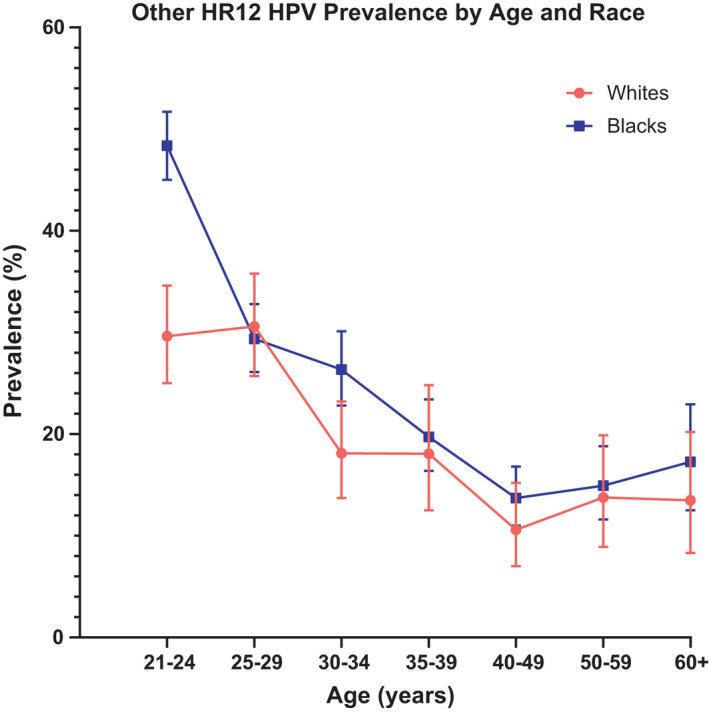
Prevalence of other HR12 HPV by age and race. The prevalence (%) and 95% confidence intervals of other HR12 HPV infection including HPV types 31, 33, 35, 39, 45, 51, 52, 56, 58, 59, 66, and 68 (*y*‐axis) are plotted by age group (*x*‐axis). Prevalence curves are shown for Whites in red with filled circles and for Blacks in blue with filled squares. HPV, human papillomavirus; HR, high‐risk

**FIGURE 3:**
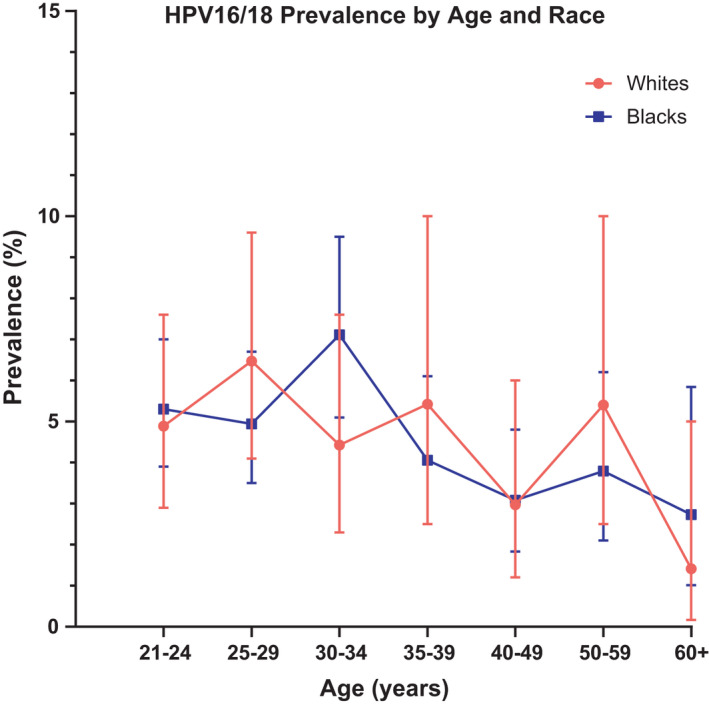
Prevalence of HPV16/18 by age and race. The prevalence (%) and 95% confidence intervals of HPV16/18 infection including HPV types 16 and 18 (*y*‐axis) are plotted by age group (*x*‐axis). Prevalence curves are shown for Whites in red with filled circles and for Blacks in blue with filled squares. HPV, human papillomavirus

Figures 2 and 3 show the prevalence of other HR12 HPV and HPV16/18 infections, respectively, by age group and race. Similar to HPV overall, the age‐specific prevalence of other HR12 HPV infections differed significantly by race among individuals aged 21–24 years, with Blacks having higher prevalence compared to Whites (48.4% vs. 29.6%, respectively; *p* < 0.0001) with the highest observed prevalence occurring between ages 25 and 29 years for Whites (30.6%) and ages 21–24 years for Blacks (48.4%). In contrast, both Whites and Blacks aged 21–24 years had very similar prevalence of HPV16/18 infections (4.9% and 5.3%, respectively), with no significant differences observed by age group. Among Whites, the highest observed prevalence of HPV16/18 infection occurred between ages 25–29 years (6.5%) and among Blacks, between ages 30–34 years (7.1%). The distribution of HPV testing and partial genotyping results by race and age were similar across both assays (data not shown).

### 3.4 Prevalence of ASC‐US/LSIL and ASC‐H/HSIL cytology by age and race

**FIGURE 4:**
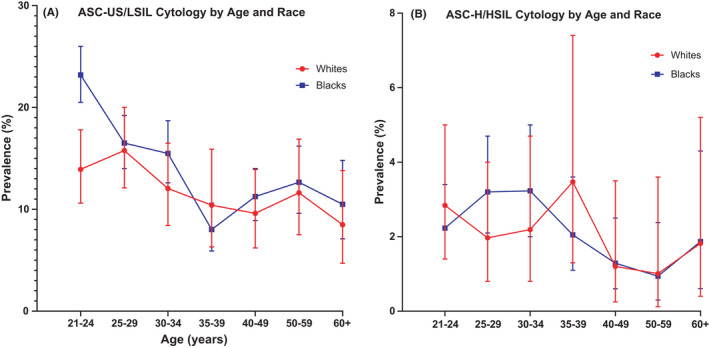
Prevalence of abnormal cytology by age and race. (A) The prevalence (%) and 95% confidence intervals of low‐grade cytology including ASC‐US and LSIL (*y*‐axis) are plotted by age group (*x*‐axis). (B) The prevalence (%) and 95% confidence intervals of high‐grade cytology including ASC‐H and HSIL (*y*‐axis) are plotted by age group (*x*‐axis). Prevalence curves are shown for Whites in red with filled circles and for Blacks in blue with filled squares. ASC‐H, atypical squamous cells cannot exclude high grade; ASC‐US, atypical squamous cells of undetermined significance; HSIL, high‐grade squamous intraepithelial lesion; LSIL, low‐grade squamous intraepithelial lesion

Blacks aged 21–24 years had significantly higher prevalence of ASC‐US/LSIL compared to Whites in the same age group (23.2% vs. 13.9%, respectively; *p* < 0.0001,  4). In contrast, while Blacks and Whites had very similar prevalence of ASC‐H/HSIL cytology at ages 21–24 years (2.2% and 2.8%, respectively), we observed an earlier peak prevalence of ASC‐H/HSIL occurring in Blacks between ages 25–29 (3.2%) compared to ages 35–39 in Whites (3.5%; Figure 4). The prevalence of ASC‐H/HSIL was similar by race at age 40 years and older.

### 3.5 Prevalence of HPV in NHANES by age and race

We observed similar HPV prevalence patterns by race in NHANES among individuals aged 21–24 years, with Blacks having higher prevalence of HPV infection overall compared to Whites (49.5%; 95% CI 38.9%–60.2% vs. 33.65%; 95% CI, 24.1–44.8%, respectively) (Figure S2). However, unlike patterns in MS, the prevalence of HPV infection was higher In Blacks compared to Whites across all age groups. Patterns were similar for other HR12 infections (Figure S3).

## 4 DISCUSSION

Racially diverse populations from settings like MS have not been well‐represented in cervical cancer research. In our baseline sample from a diverse statewide cohort of individuals undergoing screening in MS, the prevalence of carcinogenic HPV infection was 26% and was higher in Blacks (28%) compared to Whites (22%). We observed a substantial difference in the prevalence of HPV infection in the youngest age group (21–24 years), with Blacks having significantly higher prevalence compared to Whites (50.2% vs. 32.1%, respectively). While HPV16/18 prevalence was similar across racial groups, prevalence of other HR12 HPV types was significantly higher among Blacks compared to Whites. Age‐ and race‐specific prevalence of low‐grade cytologic abnormalities reflected similar patterns to those of HPV infection. With respect to high‐grade cytologic abnormalities, signaling cervical precancer, we observed an earlier peak in the prevalence in Blacks, occurring in ages 25–29, whereas this peak was observed in Whites 10 years later (ages 35–39). Collectively, our data suggest a younger age of onset of carcinogenic HPV infection and subsequent cervical precancer in Blacks compared to Whites in MS.

Given that until recently, HPV‐based screening in the United States was only recommended for women aged 30 years and older, population‐based data on racial differences in the age‐specific prevalence of HPV infection and partial genotyping are rare in individuals under 30 years of age. In general, our results were in line with NHANES data, showing higher prevalence of HPV infection overall and for other HR12 HPV types in Blacks compared to Whites aged 21–24 years, although only our study was sufficiently powered to show statistically significant differences by race. In our study, the differences in the prevalence of other HR12 HPV infections between Blacks and Whites were much less pronounced in women aged 35 years and older. In contrast, in NHANES, Blacks had higher prevalence of other HR12 HPV infections across all age groups, albeit not statistically significant. The differences between our findings in MS and NHANES seem to be related both to a higher prevalence of other HR12‐HPV infections among older Blacks in NHANES as well as higher prevalence of other HR12‐HPV infections among older Whites in MS.

It is possible that both behavioral and biological factors underlie the observed racial differences in the age‐specific HPV prevalence patterns in MS. Generally, the peak prevalence of HPV infection is tightly linked to age at sexual debut.^5^ Data from the 2013 US and MS High School Youth Risk Behavior Survey suggest an earlier age at sexual debut among Blacks compared to Whites.^21^ An important observation from our study is that differences in the prevalence of HPV infection were predominantly restricted to other HR12 HPV types. It is important to consider the effect of HPV vaccination on HPV prevalence in this context. HPV vaccination status is not reported in our data; however, MS has consistently ranked lowest in the nation for HPV vaccine coverage (29.8% in MS vs. 47.5% in the United States overall of teens aged 13–17 years up to date with HPV vaccination, 2015–2019) and Black teens are more likely to be up to date with HPV vaccination compared to White teens in MS (33.5% vs. 24.9%, respectively, 2015–2019).^22^ Given that routine HPV vaccination occurs predominantly at age 11 or 12, most vaccinated individuals in the MS population age 21 and older would have received vaccines including HPV16/18 but none of the other types. Thus, HPV vaccination likely reduced the prevalence of HPV16/18 infections both in younger Whites and Blacks, minimizing potential racial differences at younger ages. However, the striking racial differences we observed in the prevalence of other HR12 HPV infections cannot be explained by vaccination. Recent studies have shown that Blacks, based on self‐reported race and genomic ancestry informative markers, are less likely to be infected with HPV16 compared to Whites, and are more likely to be positive for other carcinogenic HPV types.^10^, ^12^, ^13^, ^14^, ^20^, ^23^, ^24^ Possible biological factors explaining these type‐specific differences by race include variation in host susceptibility and in the evolutionary “fitness” of different viral genotypes within populations of different ancestries. Implications of these type‐specific differences by race with respect to risk of cervical precancer and cancer remain unclear and require further research. In the current study, we observed earlier peaks in both other HR12 HPV positivity and high‐grade cytologic abnormalities in Blacks compared to Whites, suggesting a potential younger age of onset of cervical disease in Blacks that may be attributed to infections with non‐16/18 types. With additional follow‐up, we will be able to confirm whether these earlier peaks of high‐grade cytology are in fact associated with increased risk of cervical precancer at earlier age among Black individuals.

The data reported here represent one of the largest studies focusing on HPV natural history in a diverse, US screening population, including over 4000 Blacks. Other large population‐based screening cohorts have a much lower proportion of Black individuals,^25^ and, as we have demonstrated, HPV prevalence data based on NHANES are not well‐powered to evaluate precise age‐and‐race‐specific differences by partial genotyping. We conducted HPV testing in individuals 21–29 years of age, who are typically not represented in large clinical studies given that primary HPV screening is rarely performed under age 30. Our statewide sample covers individuals undergoing cervical cancer screening at all MS state health department clinics, with centralized data collection and pathology review. Some limitations are worth noting. We do not have information on vaccination status for individuals included in STRIDES. However, vaccination rates within the state do not appear to be differential by race in the relevant age group.^26^ We used two different assays for HPV testing, and because TypeSeq was used to supplement HPV results for those who did not receive cobas testing clinically (those who underwent primary cytology), patients with TypeSeq testing included most women younger than 30 years of age. However, TypeSeq has shown high agreement with commercial HPV assays, and we did not observe meaningful differences in the patterns by race and age by assay.^18^, ^19^ We were not able to evaluate individual genotypes in our study; however, extended genotyping of all specimens in the larger STRIDES cohort is ongoing, allowing to evaluate genotype‐specific results in the future.

Our study suggests that Blacks undergoing cervical screening in MS experience an earlier age of onset of other HR12 HPV infections and peak of high‐grade cytologic abnormalities compared to Whites. These findings may have implications for natural history modeling, risk estimation, and recommendations for HPV‐based screening. Studies of cervical carcinogenesis and screening are scarce in diverse populations like MS. STRIDES is addressing this critical gap and will continue to make important contributions to understanding HPV natural history and cervical precancer risk in a diverse high‐risk population to ensure that disease models and clinical guidelines are more inclusive and better reflect different target populations.^15^

## ETHICS STATEMENT

The Institutional Review Boards at the University of Mississippi Medical Center and Mississippi State Department of Health approved this protocol. A HIPAA waiver of authorization and HIPAA waiver of informed consent were granted.

## CONFLICT OF INTEREST

The authors declare no relevant conflict of interest.

## DISCLOSURES

The opinions or assertations contained herein are the private views of the authors and do not reflect the official policy of the Department of the Army or Department of Defense.

## Supporting information

Fig S1

Fig S2

Fig S3

Fig S3a

## Data Availability

The data that support the findings of this study are available from the corresponding author upon reasonable request.
